# Development and validation of novel models for the prediction of intravenous corticosteroid resistance in acute severe ulcerative colitis using logistic regression and machine learning

**DOI:** 10.1093/gastro/goac053

**Published:** 2022-09-30

**Authors:** Si Yu, Hui Li, Yue Li, Hui Xu, Bei Tan, Bo-Wen Tian, Yi-Min Dai, Feng Tian, Jia-Ming Qian

**Affiliations:** Department of Gastroenterology, Peking Union Medical College Hospital, Chinese Academy of Medical Sciences & Peking Union Medical College, Beijing, P. R. China; Department of Gastroenterology, Shengjing Hospital of China Medical University, Shenyang, Liaoning, P. R. China; Department of Gastroenterology, Peking Union Medical College Hospital, Chinese Academy of Medical Sciences & Peking Union Medical College, Beijing, P. R. China; Department of Gastroenterology, Peking Union Medical College Hospital, Chinese Academy of Medical Sciences & Peking Union Medical College, Beijing, P. R. China; Department of Gastroenterology, Peking Union Medical College Hospital, Chinese Academy of Medical Sciences & Peking Union Medical College, Beijing, P. R. China; Department of Gastroenterology, Peking Union Medical College Hospital, Chinese Academy of Medical Sciences & Peking Union Medical College, Beijing, P. R. China; Department of Gastroenterology, Peking Union Medical College Hospital, Chinese Academy of Medical Sciences & Peking Union Medical College, Beijing, P. R. China; Department of Gastroenterology, Shengjing Hospital of China Medical University, Shenyang, Liaoning, P. R. China; Department of Gastroenterology, Peking Union Medical College Hospital, Chinese Academy of Medical Sciences & Peking Union Medical College, Beijing, P. R. China

**Keywords:** acute severe ulcerative colitis, steroid resistance, colectomy, machine learning

## Abstract

**Background:**

The early prediction of intravenous corticosteroid (IVCS) resistance in acute severe ulcerative colitis (ASUC) patients remains an unresolved challenge. This study aims to construct and validate a model that accurately predicts IVCS resistance.

**Methods:**

A retrospective cohort was established, with consecutive inclusion of patients who met the diagnosis criteria of ASUC and received IVCS during index hospitalization in Peking Union Medical College Hospital between March 2012 and January 2020. The primary outcome was IVCS resistance. Classification models, including logistic regression and machine learning-based models, were constructed. External validation was conducted in an independent cohort from Shengjing Hospital of China Medical University.

**Results:**

A total of 129 patients were included in the derivation cohort. During index hospitalization, 102 (79.1%) patients responded to IVCS and 27 (20.9%) failed; 18 (14.0%) patients underwent colectomy in 3 months; 6 received cyclosporin as rescue therapy, and 2 eventually escalated to colectomy; 5 succeeded with infliximab as rescue therapy. The Ulcerative Colitis Endoscopic Index of Severity (UCEIS) and C-reactive protein (CRP) level at Day 3 are independent predictors of IVCS resistance. The areas under the receiver-operating characteristic curves (AUROCs) of the logistic regression, decision tree, random forest, and extreme-gradient boosting models were 0.873 (95% confidence interval [CI], 0.704–1.000), 0.648 (95% CI, 0.463–0.833), 0.650 (95% CI, 0.441–0.859), and 0.604 (95% CI, 0.416–0.792), respectively. The logistic regression model achieved the highest AUROC value of 0.703 (95% CI, 0.473–0.934) in the external validation.

**Conclusions:**

In patients with ASUC, UCEIS and CRP levels at Day 3 of IVCS treatment appeared to allow the prompt prediction of likely IVCS resistance. We found no evidence of better performance of machine learning-based models in IVCS resistance prediction in ASUC. A nomogram based on the logistic regression model might aid in the management of ASUC patients.

## Introduction

Acute severe ulcerative colitis (ASUC) is a potentially life-threatening medical emergency that requires timely recognition and intervention [1]. A total of 15%–25% of patients with ulcerative colitis (UC) will need hospitalization for an acute severe flare of disease in their natural history [2]. Intravenous corticosteroids (IVCS) are the first-line therapy for ASUC [3–5]. However, ∼30% of patients may become IVCS-resistant and require second-line therapy. Approximately 25%–30% of patients need short-term colectomy [4]. Delays in rescue therapy are associated with higher morbidity and mortality rates [6, 7]. Therefore, prompt and accurate prediction of IVCS resistance in ASUC patients is of great importance.

Many proxies have been proposed in IVCS response prediction, including the severity of clinical symptoms, laboratory biomarkers, and composite scoring systems. The erythrocyte sedimentation rate (ESR) [8, 9] and albumin (Alb) [10, 11] at Day 1 of IVCS treatment might indicate steroid failure and C-reactive protein (CRP) at Day 3 [12, 13] could predict colectomy, according to a previous study. However, conflicting results have also been reported [14–17]. The predictive value of endoscopy has been one of the research focuses, especially with the emergence of reproducible scores of endoscopic severities. However, among the widely recognized composite scoring systems in ASUC patients, such as the Travis score [17], Ho score [10], and Lindgren score [18], endoscopic features are not incorporated. There is still an unsatisfactory demand for predicting the outcomes of IVCS treatment in patients with ASUC.

Traditional approaches, such as logistic regression (LR), have long been utilized in disease outcome prediction. In the past decade, artificial intelligence has made its way into many medical domains, given the increasingly big data in electronic health records, imaging, and multiomics [19]. A recent systematic review comprehensively synthesized and appraised machine learning (ML)-based prediction models in inflammatory bowel diseases [20]. However, there is an insufficient number of ML-based models addressing the outcome prediction in patients with ASUC and the results regarding IVCS resistance are scarce [21, 22].

This study aimed to assess the predictive value of clinical, laboratory, and endoscopic parameters and develop novel predictive models for short-term outcomes in patients with ASUC. The traditional LR approach and a series of ML-based algorithms will be used in model development. External validations of the selected models will be conducted.

## Materials and methods

### Patients

This is a retrospective cohort study. A derivation cohort was recruited between March 2012 and January 2020 at Peking Union Medical College Hospital (Beijing, China). Patients who met the diagnosis criteria of ASUC (modified Truelove and Witts criteria: >6 bloody stools per day and systemic toxicity with at least one of temperature of >37.8°C, pulse of >90 bpm, hemoglobin of <105 g/L, or CRP of >30 mg/L) [3] and received IVCS (hydrocortisone at 300–400 mg/day or methylprednisolone at 60–80 mg/day) during index hospitalization were included. Patients confirmed as having Crohn’s disease during follow-up and patients with incomplete data of endoscopic and laboratory information were excluded. An independent validation cohort of the same time frame was recruited from Shengjing Hospital of China Medical University (Liaoning, China). A uniform set of criteria was used to build the two cohorts. Research approval was obtained from the Ethics Committees of Peking Union Medical College Hospital (approval no. S-K1723) and Shengjing Hospital of China Medical University (approval no. 2022PS756K). All patients provided informed consent. The study conformed with the principles in the Declaration of Helsinki.

### Predictor variables

A total of 13 demographic and clinical factors, including age, sex, duration of disease, hospital stay, stool frequency, concomitant infections, Montreal classification of disease extent [4], medication history, and extra-intestinal manifestations, were recorded during index hospitalization. Concomitant cytomegalovirus (CMV) infection was defined if CMV inclusion bodies or positive CMV-specific immunohistochemistry was identified or blood CMV DNA was detected by quantitative polymerase chain reaction (qPCR) within a week before and a week after the initiation of IVCS treatment [23]. *Clostridium difficile* infection was defined as the presence of *C. difficile* toxin A/B or the *C. difficile*-specific gene *tpi* and toxin gene (*tcdA/tcdB*) identified by polymerase chain reaction (PCR) within a week before and after the initiation of IVCS treatment [24]. Laboratory data at admission and on the third day of IVCS treatment were recorded as 14 continuous factors (absolute counts of white blood cells [WBC], neutrophils, hemoglobin level [Hgb], platelet count [PLT], CRP, ESR, and Alb). Endoscopic predictors before initiating IVCS treatment, including Mayo score, Ulcerative Colitis Endoscopic Index of Severity (UCEIS) score, luminal narrowing, and rectal sparing, were obtained through an external post hoc assessment by blinded inflammatory bowel disease (IBD) specialists.

### Definition of outcomes

The primary outcome was IVCS resistance; IVCS resistance was defined as the requirement for rescue therapy during the index hospitalization, including medical therapy and surgery. No colectomy during the index hospitalization and within 3 months after the index hospitalization, including colectomy after IVCS or after the failure of medical rescue therapy during the same exacerbation, was defined as colectomy-free.

### Statistical analysis

Statistical analysis was performed using R (Version 4.0.5; R Core Team, 2021). Continuous variables with a non-Gaussian distribution are expressed as the median and interquartile range (IQR) and were compared using the Mann–Whitney *U* test. Categorical data are presented as counts and percentages and were compared using Fisher’s exact test. Univariate analyses were first performed to identify predictors of short-term outcomes. Multivariable analysis was performed to determine the independent effects.

### Model development and validation

For the LR model, the least absolute shrinkage and selection operator (LASSO) method was used to help optimize feature selection and minimize overfitting. For the ML-based models, the package *caret* and Boruta algorithm were used for hyperparameter optimization. Models based on classifiers, including decision tree (DT), random forest (RF), and extreme-gradient boosting (XGB), were constructed. As has been published [25, 26], the discovery data set was randomly split into a 70% training set and a 30% testing set. The division procedure was replicated 100 times and the area under the receiver-operating characteristic curve (AUROC) was calculated in each split. The mean AUROC was obtained and one split with the representative AUROC that was closest to the mean AUROC was selected and presented. Predictor variable importance based on the RF model and the XGB model was trained on the entire data set.

To comprehensively evaluate the selected model, calibration curves were plotted to assess the calibration. Decision curve analysis was conducted to determine the clinical usefulness and net benefit. A predicting nomogram of the LR model was eventually established. The external validity of the selected models was confirmed with data from the validation cohort.

## Results

### Patient characteristics

A total of 196 patients with UC were hospitalized and screened. Among these patients, 2 were diagnosed with Crohn’s colitis during follow-up and 31 were excluded for lack of endoscopic and laboratory data; 34 patients did not rigorously meet the modified Truelove and Witts criteria for ASUC. Eventually, 129 patients with ASUC were included in the analysis (Table 1). The median age at admission was 40 years (IQR 30–51). Sixty-two (48.1%) patients were female. Twelve (9.3%) patients were newly diagnosed with UC and the others suffered from recurrence. The median duration of UC was 2 years (IQR 1.0–6.0). A total of 121 (93.8%) patients had extensive UC (E3) and 8 (6.2%) patients had left-sided UC (E2). Sixty-nine (53.5%) of the patients had received treatments for UC before admission, including oral or systemic corticosteroids (67, 51.9%), immunosuppressants (10, 7.8%), and biologics (7, 5.4%). Of the seven patients who had been treated with biologics before hospitalization, two received adalimumab and five received infliximab. Six cases of extra-intestinal manifestations at admission were reported, including primary biliary cholangitis (*n *=* *1), ankylosing spondylitis (*n *=* *2), and venous thrombosis (*n *=* *3: 2 cases of upper extremity superficial vein thrombosis and 1 case of lower extremity inter-muscular vein thrombosis).

**Table 1.** Characterization of the acute severe ulcerative colitis patient cohort

Characteristic	IVCS response	IVCS resistance	*P*-value
	*n = *102	*n = *27	
Female, *n* (%)	48 (47.1)	14 (51.9)	0.821
Age, median (IQR), years	38.5 (28.2–49.8)	46.0 (35.0–56.5)	0.079
Duration of disease, median (IQR), years	3.0 (1.0–7.0)	1.0 (0.5–3.0)	0.071
Stool frequency, median (IQR)	10.0 (8.0–15.0)	10.0 (8.0–15.5)	0.807
Extra-intestinal manifestation, *n* (%)	4 (3.9)	2 (7.4)	0.605
Duration of IVCS, median (IQR), days	10.5 (7.0–14.0)	14.0 (11.0–20.5)	0.004
Hospital stay, median (IQR), days	24.0 (17.2–30.0)	43.0 (37.0–51.0)	<0.001
*Clostridium difficile* infection, *n* (%)	2 (2.0)	2 (7.4)	0.193
Cytomegalovirus infection, *n* (%)	15 (14.7)	7 (25.9)	0.247
Laboratory tests at admission, median (IQR)			
CRP, mg/L	45.8 (23.8–79.7)	63.0 (38.7–122.0)	0.041
ESR, mm/h	40.0 (23.0–57.5)	39.0 (22.5–59.5)	0.824
Alb, g/L	30.0 (27.0–33.8)	29.0 (25.0–32.0)	0.255
Laboratory tests at Day 3 of IVCS, median (IQR)			
CRP, mg/L	8.8 (4.2–19.7)	34.0 (15.2–60.4)	<0.001
ESR, mm/h	21.0 (15.0–36.2)	26.0 (16.5–37.5)	0.578
Alb, g/L	29.0 (26.2–32.0)	28.0 (26.0–30.0)	0.245
Medical history, *n* (%)			
Steroid use	49 (48.0)	18 (66.7)	0.132
Immunosuppressant	7 (6.9)	3 (11.1)	0.436
Biologics	6 (5.9)	1 (3.7)	1.000
Endoscopic performance			
Mayo score = 3, *n* (%)	82 (80.4)	27 (100)	0.007
UCEIS scores, median (IQR)	6.00 (5.25–7.00)	7.00 (7.00–8.00)	<0.001
Lumen narrowing, *n* (%)	24 (23.5)	8 (29.6)	0.688
Rectal sparing, *n* (%)	30 (29.4)	8 (29.6)	1.000
Montreal classification of disease extent, *n* (%)			0.203
E2	8 (7.8)	0 (0)	
E3	94 (92.2)	27 (100)	

IVCS, intravenous corticosteroid; IQR, interquartile range; CRP, C-reactive protein; ESR, erythrocyte sedimentation rate; Alb, albumin; UCEIS, Ulcerative Colitis Endoscopic Index of Severity; E2, left-sided ulcerative colitis; E3, pancolitis.

**Figure 1.**
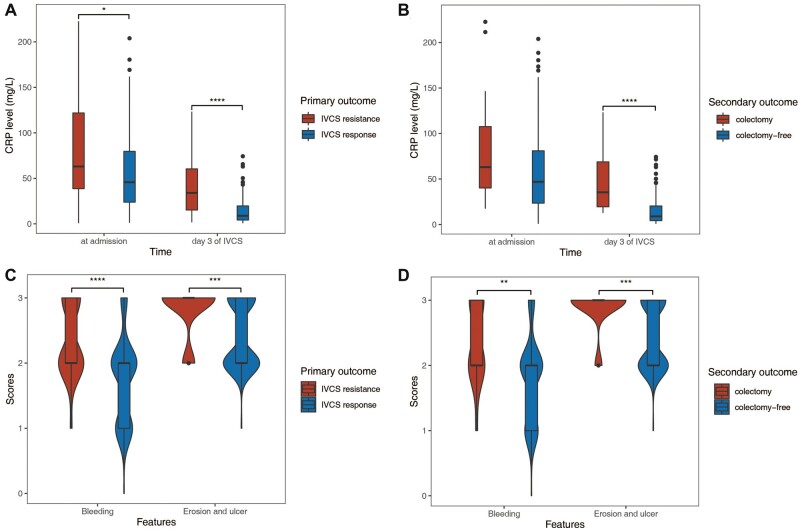
Detailed analysis of CRP levels and endoscopic features in ASUC patients. (A) and (B) CRP levels at different periods of IVCS treatment. (C) and (D) Violin plot analysis comparing the distribution of endoscopic severity represented by UCEIS descriptors in patients with different clinical outcomes. *P <* *0.05; **P <* *0.01; ***P <* *0.001; ****P <* *0.0001. CRP, C-reactive protein; ASUC, acute severe ulcerative colitis; IVCS, intravenous corticosteroid.

Compared with patients who were eventually resistant to IVCS, the IVCS responders had a shorter duration of IVCS use (10.5 [7.0–14.0] vs 14.0 [11.0–20.5] days, *P *=* *0.004) and hospital stay (24.0 [17.2–30.0] vs 43.0 [37.0–51.0] days, *P *<* *0.001). The CRP levels at admission (45.8 [23.8–79.7] vs 63.0 [38.7–122.0] mg/L, *P *=* *0.041) and on the third day of IVCS treatment (8.8 [4.2–19.7] vs 34.0 [15.2–60.4] mg/L, *P *<* *0.001, Figure 1A and B) were significantly higher in the non-responders, as well as the severity scores of endoscopic performances, including the Mayo scores (percentage of Mayo scores = 3, 80.4% vs 100%, *P *=* *0.007) and UCEIS scores (6.00 [5.25–7.00] vs 7.00 [7.00–8.00] points [pts], *P *<* *0.001; Table 1). Distinct patterns of endoscopic features were observed in patients with different outcomes (Figure 1C and D). Of the three descriptive factors constituting the UCEIS score, vascular patterns were similar between groups, whereas bleeding and erosions and ulcers were more severe in IVCS-resistant patients and patients who needed colectomy. Full characterization of the cohort is provided in Supplementary Table 1.

### Clinical outcomes

During index hospitalization, 102 (79.1%) ASUC patients responded to IVCS, whereas 27 (20.9%) patients were resistant to IVCS. The rescue therapy was cyclosporin in 6 patients, infliximab in 5 patients, and colectomy in 16 (12.4%) patients. Two of the patients treated with cyclosporin eventually needed colectomy. Patients treated with infliximab were all free of colectomy. The colectomy rate within 3 months after admission was 14.0% (*n *=* *18), with a median duration of 28 (IQR, 20.0–34.0) days.

### Predictors of short-term outcomes

Univariate analysis was performed to determine factors that were associated with IVCS resistance within 3 months. PLT at admission, serum level of CRP at admission and Day 3 of IVCS treatment, prior steroid use, and UCEIS scores were potential factors associated with IVCS resistance within 3 months (all *P *<* *0.1; Table 2; univariate analysis of all the factors shown in Supplementary Table 2). Patients who received colectomy within 3 months were significantly older at the episode of ASUC (*P *=* *0.033). By multivariate analysis, UCEIS score (odds ratio [OR], 5.67; 95% confidence interval [CI], 2.34–13.72, *P *<* *0.001) and CRP at Day 3 of IVCS treatment (OR, 1.05; 95% CI, 1.02–1.08, *P *=* *0.001) were identified as independent predictors of IVCS resistance.

**Table 2.** Univariate and multivariate analyses of possible predictors of intravenous corticosteroid resistance

Characteristic	Univariate analysis			Multivariate analysis		
	OR	95% CI	*P*-value	OR	95% CI	*P*-value
PLT at admission, × 10^9^/L	1.00	0.99–1.00	0.05	1.00	0.99–1.00	0.18
CRP at admission, mg/L	1.01	1.00–1.02	0.02	1.00	0.99–1.01	0.76
CRP at Day 3 of IVCS, mg/L	1.05	1.03–1.07	<0.001	1.05	1.02–1.08	0.001
Prior steroid use, yes vs no	2.16	0.89–5.27	0.09	2.49	0.76–8.18	0.13
UCEIS scores	5.44	2.50–11.85	<0.001	5.67	2.34–13.72	<0.001

PLT, platelet; CRP, C-reactive protein; IVCS, intravenous corticosteroid; UCEIS, Ulcerative Colitis Endoscopic Index of Severity; OR, odds ratio; CI, confidence interval.

### Existing scoring system and clinical outcomes

Three widely recognized indices for patients with ASUC, the Travis [17], Ho [10], and Lindgren [18] scores, were calculated (Supplementary Table 3). There was no significant difference in Travis and Ho scores between IVCS responders and IVCS non-responders. However, patients with steroid failure had significantly higher Lindgren scores (15.97 [10.76–25.26] vs 11.50 [9.39–18.92], *P *=* *0.018). The AUROC values of the Travis, Ho, and Lindgren scores were 0.526 (95% CI, 0.337–0.715), 0.587 (95% CI, 0.374–0.800), and 0.561 (95% CI, 0.371–0.751), respectively.

### Development and evaluation of the novel predictive models

#### Development of the predictive models

**Figure 2.**
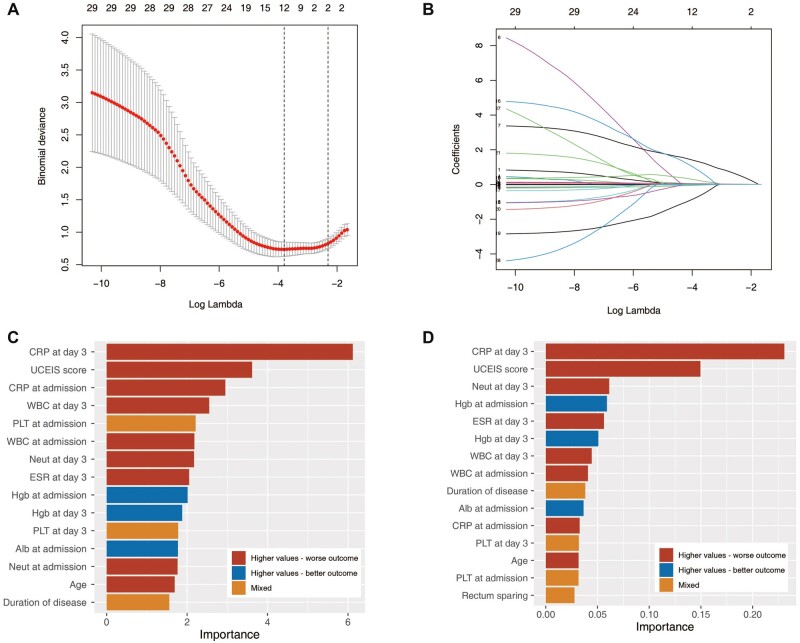
Predictors and feature selection. (A) Optimal number of parameters (lambda) determination in the least absolute shrinkage and selection operator (LASSO) model using 5-fold cross-validation via 1 standard error (SE) of minimum criteria. The binomial deviance curve was plotted against log(lambda). Vertical dotted lines indicate the optimal value of the minimum criteria and 1 SE of the minimum criteria. (B) Coefficient profile of the 27 parameters was plotted vs log(lambda). 1 SE minimum criteria corresponded to two nonzero coefficients. (C) The top 15 important predictors in the random forest model. (D) The top 15 important predictors in the extreme-gradient boosting model. The higher the importance, the larger the discrimination provided by the predictor. CRP, C-reactive protein; UCEIS, Ulcerative Colitis Endoscopic Index of Severity; WBC, white blood cell; PLT, platelet; Neut, neutrophil; ESR, erythrocyte sedimentation rate; Hgb, hemoglobin; Alb, albumin.

A total of 28 variables were reduced to 2 potential predictors by LASSO regression analysis (Figure 2A and B), UCEIS, and CRP at Day 3 of IVCS treatment, which were incorporated into the LR model (β0 = −12.871, β for UCEIS = 1.543, β for CRP at Day 3 = 0.053). Three features, including CRP at Day 3 of >34.6 mg/L, UCEIS score of >6.5 pts, and CRP at admission of >103.1 mg/L, entered the DT model. The top five important predictors in the RF model were CRP at Day 3, UCEIS score, CRP at admission, WBC at Day 3, and PLT at admission (Figure 2C). The top five important predictors in the XGB model were CRP at Day 3, UCEIS score, neutrophils at Day 3, Hgb at admission, and ESR at Day 3 (Figure 2D).

#### Validation of the predictive models

**Figure 3.**
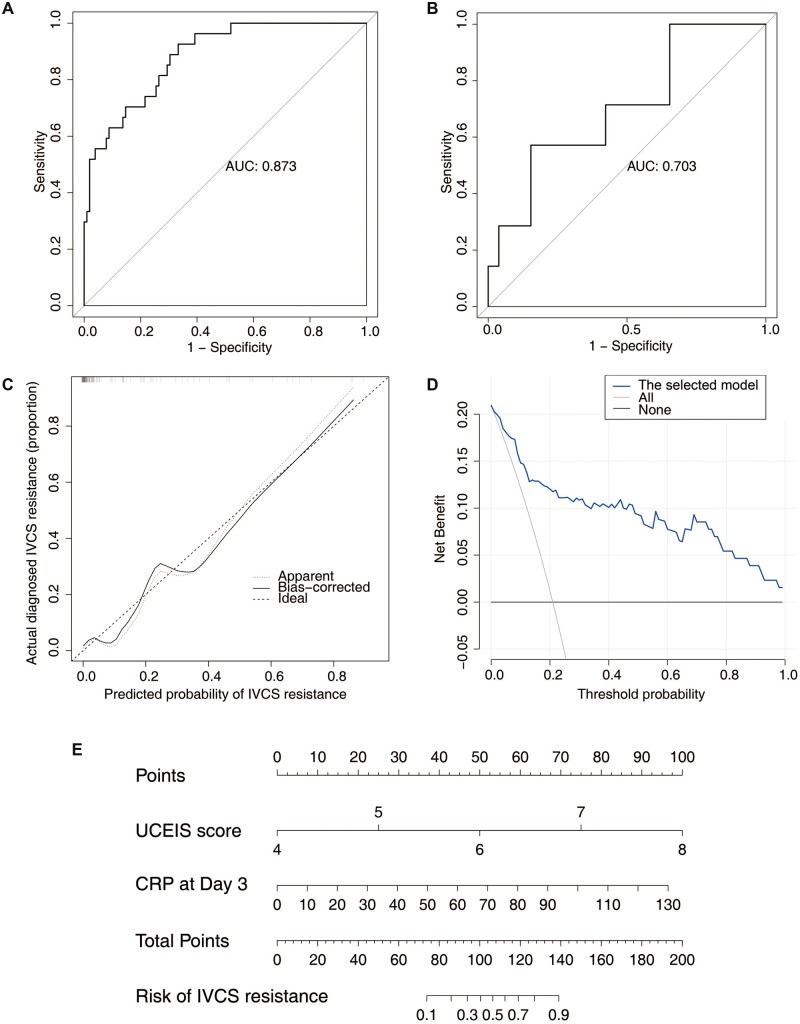
Pragmatic model and nomogram. (A) and (B) ROC curves of the pragmatic model in the derivation and validation cohorts. (C) Calibration curves of the pragmatic model prediction. Perfect prediction by an ideal model is represented by the diagonal dotted line. (D) Decision curve of the pragmatic model. The gray and black curves represent the clinical benefit of rescue therapy in all patients and none, respectively. (E) Predictive nomogram of the pragmatic model. ROC, receiver-operating characteristic; AUC, area under the ROC curve; UCEIS, Ulcerative Colitis Endoscopic Index of Severity; CRP, C-reactive protein; IVCS, intravenous corticosteroid.

In the internal validation, the representative AUROC values of the LR, DT, RF, and XGB models were 0.873 (95% CI, 0.704–1.000; Figure 3A), 0.648 (95% CI, 0.463–0.833), 0.650 (95% CI, 0.441–0.859), and 0.604 (95% CI, 0.416–0.792), respectively (Table 3). Sixty-five ASUC patients were included in the external validation cohort, with a median age of 44 (IQR, 31.5–52.5) years (Supplementary Table 4). The proportions of IVCS resistance and colectomy within 3 months were 21.5% (*n *=* *14) and 3.1% (*n *=* *2), respectively. The median UCEIS scores and median CRP levels at Day 3 were 5 pts (IQR 4–6) and 11.6 mg/L (IQR 5.9–25.7), respectively. The AUROC values of each model in the validation cohort are shown in Table 3. The LR model achieved the highest AUROC value of 0.703 (95% CI, 0.473–0.934) in the external validation (Table 3 and Figure 3B).

**Table 3.** Performance of the LR model and the ML-based models

Model	Internal validation		External validation	
	AUROC	95% CI	AUROC	95% CI
Logistic regression	0.873	0.704–1.000	0.703	0.473–0.934
Decision tree	0.648	0.463–0.833	0.514	0.360–0.667
Random forest	0.650	0.441–0.859	0.552	0.407–0.697
Extreme-gradient boosting	0.604	0.416–0.792	0.585	0.394–0.776

AUROC, area under the receiver-operating characteristic curve; CI, confidence interval.

#### Pragmatic model and nomogram

Synthetically considered among the predictive performances and clinical availability, the LR model was selected as the final model for IVCS resistance prediction. The calibration curve of the LR model demonstrated good agreement in the retrospective cohort (Figure 3C). As presented in Figure 3D, the decision curve analysis graphically shows the clinical usefulness of the predicting model based on a continuum of potential thresholds for IVCS resistance risk (*x*-axis) and the net benefit of using the model to risk stratify patients (*y*-axis) relative to assuming that no patient will be IVCS-resistant. A nomogram based on the selected model was constructed (Figure 3E).

## Discussion

Accurate risk assessment of IVCS resistance in patients with ASUC is paramount since delaying the initiation of rescue therapy is a recognized determinant of morbidity and mortality [6]. Biomarkers and composite scoring systems might help predict outcomes in ASUC. However, the roles of individual biomarkers require further clarification and there remains considerable uncertainty over the utility and preferences of the scoring system [27]. An effective predictive tool could help physicians identify patients requiring escalation to reduce unnecessary steroid exposure and improve prognosis.

In this study, we used the traditional LR model and ML approaches to generate predictive models of IVCS resistance in patients with ASUC. The LR model showed good accuracy in internal validation. In the validation cohort, the LR model showed an AUROC of 0.703 (95% CI, 0.473–0.934), suggesting satisfactory generalization capability. The DT, RF, and XGB models, by contrast, performed worse and were relatively inaccurate in both the internal and external validation. In addition, the LR model showed better performance than the Travis, Ho, and Lindgren scores. All three scores incorporate stool frequency, which could be inaccurate due to information bias. In addition, none of the scores incorporated endoscopic findings, which are clearly a parameter worthy of careful consideration as a predictive tool. In consideration of model performance and clinical utility, the LR model was selected as the final pragmatic model. Eventually, a nomogram was established for convenient individualized risk stratification and clinical decision-making in patients with ASUC.

The application of artificial intelligence in medicine has been a hot topic recently. Nguyen *et al.* [20] comprehensively analysed 13 ML-based prediction models in the field of IBD and concluded that ML models generally perform better than traditional statistical models. However, the flaws in these models lie in the lack of external validation and clinical applicability. Christodoulou *et al.* [19] identified 282 comparisons between LR and ML models and found that there was no significant difference in performance for comparisons at low risk of bias. The conflict and our results can be interpreted from the following aspects. First, ML models tend to perform well in problems with a strong signal-to-noise ratio [28], but clinical prediction problems usually have a low signal-to-noise ratio. Second, class imbalance is a common problem in ML model development, but adjusting class imbalance could distort prevalence, which is not appropriate in clinical scenarios [29]. Finally, as suggested by previous research, compared with LR models, ML models need more data to achieve an ideal prediction [30, 31]. Indeed, for high-dimensional data, such as multiomics and imaging data, ML-based approaches may be good choices. However, in the scenario of this study, the LR model was better. It does not negate the usability of ML-based approaches; with the rapid growth of clinical data, ML-based models still hold great potential for medical application and further exploration is warranted.

The UCEIS score and CRP level at Day 3 of IVCS treatment were demonstrated to be two crucial predictors of IVCS resistance in patients with ASUC. The UCEIS score and CRP level were identified as the only two independent predictors in the multivariate analysis. These two factors also had the highest importance in the ML-based models. Our findings regarding the UCEIS score indicated that it outperformed the Mayo score in risk stratification and prediction. We also found that ASUC patients who were eventually resistant to corticosteroids and received colectomy were more likely to have luminal bleeding (2–3 pts) and deep ulcers (3 pts). These findings were consistent with previous studies supporting the predictive value of UCEIS in predicting steroid failure [13, 32, 33]. Therefore, we suggest careful and detailed examination and evaluation of endoscopic characteristics during the hospital index of patients with ASUC. Regarding the CRP levels, our results were consistent with previous studies showing that the CRP level at Day 3 of IVCS treatment was predictive of steroid failure [10, 17, 18, 34]. Generally, the importance of inflammatory marker monitoring in the early stage of IVCS treatment should be emphasized.

The study has several limitations. First, the small sample size from a single center makes it difficult to inform the effects of some factors. However, our sample size is comparable to the sample size in the original study of Travis and Ho scores [10, 17], which are widely accepted. In addition, we included an independent validation cohort that helped determine the reproducibility and generalizability in different patients [35]. Nevertheless, the IVCS resistance rates in the validation cohort were slightly higher and the colectomy rates were lower than those in the derivation cohort, which may induce miscalibration. In the future, the model can be modified by adjusting the baseline hazard or model intercept to better suit the average outcome risk in a larger external population [35]. Second, endoscopic features and laboratory data are currently routinely involved in the diagnosis and decision-making of clinicians, which could introduce circularity of argument, an inevitable inherent issue in data interpretation, and a prospective study may help in the future [27]. Moreover, in addition to endoscopic features and laboratory data, many novel predictors for IVCS response in UC patients have been proposed, including fecal calprotectin levels [36, 37] and histological indices [38–40]. These factors were not recorded in our study due to restrictions of objective conditions. The predictive value of these factors and a composite score incorporating these factors need to be assessed in the future.

In conclusion, this study has developed and validated novel models to predict IVCS resistance in patients with ASUC. The UCEIS score and CRP levels at Day 3 of IVCS treatment were defined as factors that dramatically influenced the short-term outcome. ML did not show better performance than the traditional LR model in this scenario. The pragmatic model might help in the management of patients with ASUC.

## Supplementary Data

Supplementary data is available at *Gastroenterology Report* online.

## Authors’ Contributions

Y.L., F.T., and J.M.Q. conceived and designed the project. H.L., B.W.T., and Y.M.D. collected the data. S.Y., H.X., and B.T. analysed and interpreted the data. S.Y. drafted the manuscript. All authors have read and approved the final version of the manuscript.

## Funding

This work was supported by the Beijing Municipal Natural Science Foundation [grant number 7212078] and the CAMS Innovation Fund for Medical Sciences (CIFMS) [grant number 2020-I2 M-C&T-B-005].

## Conflict of Interest

None declared.

## Supplementary Material

goac053_Supplementary_Data
