# Supplementary material for: Development and validation of novel models for the prediction of intravenous corticosteroid resistance in acute severe ulcerative colitis using logistic regression and machine learning
Source: Gastroenterol Rep (Oxf). 2022 Sep 30;10:goac053. doi: 10.1093/gastro/goac053 (PMC9525078; doi:10.1093/gastro/goac053)
Supplement: goac053_Supplementary_Data [file goac053_supplementary_data.docx]

**Supplementary Table 1.** Comparison of IVCS response and IVCS resistance patients in the derivation cohort

| Characteristic | Total  *n =* 129 | IVCS response  *n =* 102 | IVCS resistance  *n =* 27 | *P* value |
| --- | --- | --- | --- | --- |
| Female, *n* (%) | 62 (48.1) | 48 (47.1) | 14 (51.9) | 0.821 |
| Age, median (IQR), years | 40.0 (30.0–51.0) | 38.5 (28.2–49.8) | 46.0 (35.0–56.5) | 0.079 |
| Duration of disease, median (IQR), years | 2.0 (1.0–6.0) | 3.0 (1.0–7.0) | 1.0 (0.5–3.0) | 0.071 |
| Stool frequency at admission, median (IQR) | 10.0 (8.0–15.0) | 10.0 (8.0–15.0) | 10.0 (8.00–15.5) | 0.807 |
| Extraintestinal manifestation, *n* (%) | 6 (4.7) | 4 (3.9) | 2 (7.4) | 0.605 |
| Duration of IVCS, median (IQR), days | 11.0 (8.0–15.0) | 10.5 (7.0–14.0) | 14.0 (11.0–20.5) | 0.004 |
| Hospital stay, median (IQR), days | 27.0 (19.0–41.0) | 24.0 (17.2–30.0) | 43.0 (37.0–51.0) | < 0.001 |
| *Clostridium difficile* infection, *n* (%) | 4 (3.1) | 2 (2.0) | 2 (7.4) | 0.193 |
| Cytomegalovirus infection, *n* (%) | 22 (17.1) | 15 (14.7) | 7 (25.9) | 0.247 |
| Laboratory tests at admission |  |  |  |  |
| White blood cell count, median (IQR), × 10^9^/L | 8.51 (6.04–10.7) | 8.86 (6.34–10.9) | 7.31 (5.89–9.96) | 0.347 |
| Neutrophils, median (IQR), × 10^9^/L | 5.73 (4.14–8.14) | 5.73 (3.97–8.13) | 5.46 (4.51–7.85) | 0.880 |
| Hemoglobin, mean ± SD, g/L | 99.8 ± 24.9 | 101 ± 25.3 | 95.3 ± 23.5 | 0.199 |
| Platelet count, median (IQR), × 10^9^/L | 341 (267–437) | 342 (268–444) | 327 (260–389) | 0.152 |
| C-reactive protein, median (IQR), mg/L | 51.4 (24.7–86.3) | 45.8 (23.8–79.7) | 63.0 (38.7–122.0) | 0.041 |
| Erythrocyte sedimentation rate, median (IQR), mm/h | 40.0 (23.0–59.0) | 40.0 (23.0–57.5) | 39.0 (22.5–59.5) | 0.824 |
| Albumin, median (IQR), g/L | 30.0 (27.0–33.0) | 30.0 (27.0–33.8) | 29.0 (25.0–32.0) | 0.255 |
| Laboratory tests at Day 3 of IVCS, median (IQR) |  |  |  |  |
| White blood cell count, × 10^9^/L | 8.39 (6.21–10.3) | 8.40 (6.60–10.4) | 7.72 (5.40–10.00) | 0.376 |
| Neutrophils, × 10^9^/L | 5.54 (4.20–7.65) | 5.54 (4.21–7.57) | 5.66 (4.22–8.00) | 0.908 |
| Hemoglobin, g/L | 92.0 (80.0–107) | 94.0 (80.2–108) | 85.0 (80.0–93.5) | 0.131 |
| Platelet count, × 10^9^/L | 335 (261–432) | 338 (259–450) | 309 (272–382) | 0.384 |
| C-reactive protein, mg/L | 12.5 (4.9–25.4) | 8.8 (4.2–19.7) | 34.0 (15.2–60.4) | < 0.001 |
| Erythrocyte sedimentation rate, mm/h | 23.0 (15.0–37.0) | 21.0 (15.0–36.2) | 26.0 (16.5–37.5) | 0.578 |
| Albumin, median (IQR), g/L | 28.0 (26.0–32.0) | 29.0 (26.2–32.0) | 28.0 (26.0–30.0) | 0.245 |
| Prior steroid use, *n* (%) | 67 (51.9) | 49 (48.0) | 18 (66.7) | 0.132 |
| Prior immunosuppressant use, *n* (%) | 10 (7.8) | 7 (6.9) | 3 (11.1) | 0.436 |
| Prior biologics use, *n* (%) | 7 (5.4) | 6 (5.9) | 1 (3.7) | 1.000 |
| Mayo scores = 3, *n* (%) | 109 (84.5) | 82 (80.4) | 27 (100) | 0.007 |
| UCEIS scores, median (IQR) | 7.00 (6.00–7.00) | 6.00 (5.25–7.00) | 7.00 (7.00–8.00) | < 0.001 |
| Bleeding scores, median (IQR) | 2.00 (1.00–2.00) | 2.00 (1.00–2.00) | 2.00 (2.00–3.00) | < 0.001 |
| Ulcer and erosion scores, median (IQR) | 3.00 (2.00–3.00) | 2.00 (2.00–3.00) | 3.00 (3.00–3.00) | < 0.001 |
| Lumen narrowing, *n* (%) | 32 (24.8) | 24 (23.5) | 8 (29.6) | 0.688 |
| Rectal sparing, *n* (%) | 38 (29.5) | 30 (29.4) | 8 (29.6) | 1.000 |
| Montreal classification of disease extent, *n* (%) |  |  |  | 0.203 |
| E2 | 8 (6.2) | 8 (7.8) | 0 (0) |  |
| E3 | 121 (93.8) | 94 (92.2) | 27 (100) |  |
| Colectomy within 3 months, *n* (%) | 18 (14.0) | - | 18 (66.7) | - |

IVCS, intravenous corticosteroid; UCEIS, Ulcerative Colitis Endoscopic Index of Severity; E2, left-sided ulcerative colitis; E3, pancolitis.

**Supplementary Table 2.** Univariate analysis of potential predictors

| Characteristic | Odd ratio | 95% CI | *P* value |
| --- | --- | --- | --- |
| Age, years | 1.02 | 1.00–1.05 | 0.10 |
| Duration of IVCS, days | 0.94 | 0.85–1.04 | 0.22 |
| *Clostridium difficile* infection | 4.00 | 0.54–29.81 | 0.18 |
| Cytomegalovirus infection | 2.03 | 0.73–5.63 | 0.17 |
| Mayo score = 3 vs Mayo score < 3 | 6.34 | 0.81–49.57 | 0.08 |
| UCEIS scores | 5.44 | 2.50–11.85 | < 0.001 |
| WBC at admission, × 10^9^/L | 1.01 | 1.00–1.01 | 0.23 |
| Neut, × 10^9^/L | 1.06 | 0.92–1.22 | 0.42 |
| Hgb at admission, g/L | 0.99 | 0.97–1.01 | 0.30 |
| PLT at admission, × 10^9^/L | 1.00 | 0.99–1.00 | 0.05 |
| CRP at admission, mg/L | 1.01 | 1.00–1.02 | 0.02 |
| ESR at admission, mm/h | 1.00 | 0.98–1.01 | 0.65 |
| Alb at admission, g/L | 0.94 | 0.87–1.03 | 0.18 |
| Prior steroid use | 2.16 | 0.89–5.27 | 0.09 |
| Prior immunosuppressant use | 1.70 | 0.41–7.05 | 0.47 |
| Prior biologics use | 0.62 | 0.07–5.34 | 0.66 |
| Lumen narrowing | 1.37 | 0.53–3.52 | 0.52 |
| Rectal sparing | 1.01 | 0.40–2.56 | 0.98 |
| d3 WBC, × 10^9^/L | 0.98 | 0.86–1.12 | 0.82 |
| d3 Neut, × 10^9^/L | 1.07 | 0.93–1.23 | 0.34 |
| d3 Hgb, g/L | 0.98 | 0.95–1.00 | 0.10 |
| d3 PLT, × 10^9^/L | 1.00 | 0.99–1.00 | 0.18 |
| d3 CRP, mg/L | 1.05 | 1.03–1.07 | < 0.001 |
| d3 ESR, mm/h | 1.01 | 0.98–1.03 | 0.54 |
| d3 Alb, g/L | 0.94 | 0.84–1.05 | 0.25 |
| Stool frequency | 1.02 | 0.96–1.08 | 0.53 |
| Gender | 1.21 | 0.52–2.83 | 0.66 |

IVCS, intravenous corticosteroid; WBC, white blood cells; Neut, neutrophils; Hgb, hemoglobin; PLT, platelet; CRP, C-reactive protein; ESR, erythrocyte sedimentation rate; Alb, albumin; UCEIS, Ulcerative Colitis Endoscopic Index of Severity; d3, at Day 3 of IVCS treatment; CI, confidence interval.

**Supplementary Table 3.** Existing scoring system and clinical outcomes

|  | Travis score | Ho score | Lindgren score |
| --- | --- | --- | --- |
| Criteria/formula | High risk: stool frequency > 8/d or 3–8/d with a CRP > 45 mg/L on the third day of admission | Stool frequency on the third day of admission (< 4/d = 0 points, 4–6/d = 1 point, 6–9/d = 2 points, > 9/d = 4 points) + hypoalbuminemia at admission (< 30 g/L = 1 point) + colonic dilation on abdominal X-ray at the third day of admission (> 5.5 cm = 4 points) | Stool frequency/d + 0.14 × CRP on the third day of admission |
| Comparison between patients with different clinical outcomes | | | |
| IVCS response (*n* = 102) | 67 (65.7%) | 4.00 (3.00–4.25) | 11.50 (9.39–18.92) |
| IVCS resistance (*n* = 27) | 20 (74.1%) | 4.00 (2.00–5.00) | 15.97 (10.76–25.26) |
| *P* value | 0.493 | 0.813 | 0.018 |
| Univariate analysis | | | |
| Odd ratio | 1.493 | 1.049 | 1.068 |
| *P* value | 0.410 | 0.792 | 0.010 |
| Predictive performance | | | |
| AUROC | 0.526 | 0.587 | 0.561 |
| 95% CI | 0.337–0.715 | 0.374–0.800 | 0.371–0.751 |

CRP, C-reactive protein; IVCS, intravenous corticosteroid; AUROC, area under the receiver operating characteristic curve; CI, confidence interval.

**Supplementary Table 4.** Characteristics of the external validation cohort

| Characteristic | Total  *n* = 65 | IVCS response  *n* = 51 | IVCS resistance  *n* = 14 |
| --- | --- | --- | --- |
| Age, median (IQR), years | 44.0 (31.5–52.5) | 42.0 (28.8–52.3) | 49.0 (42.0–54.0) |
| UCEIS score, median (IQR) | 5.00 (4.00–6.00) | 5.00 (4.00–6.00) | 6.00 (4.00–6.00) |
| CRP at admission, median (IQR), mg/L | 72.6 (34.4–110.0) | 73.2 (36.2–109.5) | 54.4 (29.6–163.0) |
| CRP at Day 3 of IVCS, median (IQR), mg/L | 11.6 (5.9–25.7) | 10.56 (5.5–22.3) | 16.0 (10.2–32.1) |
| Prior steroid use, *n* (%) | 4 (6.2) | 4 (7.8) | 0 (0) |
| Colectomy within 3 months, *n* (%) | 2 (3.1) | - | 2 (14.3) |

IVCS, intravenous corticosteroid; UCEIS, Ulcerative Colitis Endoscopic Index of Severity; CRP, C-reactive protein.
